# Spontaneous coronary artery dissection: a focus on post-dissection care for the vascular medicine clinician

**DOI:** 10.3389/fcvm.2024.1352700

**Published:** 2024-03-20

**Authors:** Katherine A. Martinez, Brent Gudenkauf, Elizabeth V. Ratchford, Esther S. H. Kim, Garima Sharma

**Affiliations:** ^1^ Biochemistry Undergraduate Program, Loyola University Maryland, Baltimore, MD, United States; ^2^ The Texas Heart Institute Fellowship Program, Houston, TX, United States; ^3^ Johns Hopkins Center for Vascular Medicine, Johns Hopkins University School of Medicine, Baltimore, MD, United States; ^4^ Sanger Heart and Vascular Institute, Atrium Health, Charlotte NC, United States; ^5^ Inova Schar Heart and Vascular, Inova Fairfax Medical Campus, Falls Church, VA, United States

**Keywords:** SCAD, ACS, pregnancy, acute myocardial infarction, pregnancy associated sudden coronary artery dissection

## Abstract

Spontaneous coronary artery dissection (SCAD) is an uncommon condition which is increasingly recognized as a cause of significant morbidity. SCAD can cause acute coronary syndrome and myocardial infarction (MI), as well as sudden cardiac death. It presents similarly to atherosclerotic MI although typically in patients with few or no atherosclerotic risk factors, and particularly in women. As more patients are recognized to have this condition, there is a great need for clinician familiarity with diagnostic criteria, as well as with contemporary treatment approaches, and with appropriate patient-centered counseling, including genetic testing, exercise recommendations, and psychological care. The standard of care for patients with SCAD is rapidly evolving. This review therefore summarizes the diagnosis of SCAD, epidemiology, modern treatment, cardiac rehabilitation and preconception counseling, and the current approach to genetic testing, exercise recommendations, and psychological care, all of which are crucial to the vascular medicine specialist.

## Introduction

Spontaneous coronary artery dissection (SCAD) was first recognized in 1931 (1) and is an uncommon but important cause of myocardial infarction (MI), particularly in young people (1). It causes less than 1% of MI in some cohorts although it is likely underdiagnosed despite recent efforts at increasing awareness (2). SCAD occurs primarily in women who may not have traditional risk factors for atherosclerotic coronary artery disease and causes 25%–30% of MI in women younger than 50 years of age, as well as 15%–20% of peripartum and pregnancy-associated MI (3–7). Its pathophysiology is incompletely understood, but it probably results from dissection of the tunica media or rupture of the vasavasorum, causing intramural hemorrhage and hematoma and resultant luminal obstruction by the dissection flap or the hematoma itself. This may result in ischemia or infarction (8, 9). Prompt diagnosis is essential, as treatment differs considerably from the more common causes of MI, and SCAD is associated with high rates of complications (2). Further, SCAD highlights a vascular system at risk of further dissection, and it is linked to other vasculopathies such as fibromuscular dysplasia (FMD) and aneurysms (10, 11). A diagnosis should prompt diagnostic imaging of other vascular territories to identify aneurysms or extra-coronary abnormalities requiring treatment and referral to a vascular specialist (12). Patients may also benefit from lifestyle counseling to decrease the risk of recurrence (12). This review will discuss the clinical manifestations and various types of SCAD, diagnostic findings, surveillance for disease in the non-coronary vasculature, pharmacologic management, genetic testing, and advice for patients, particularly for patients considering pregnancy. As SCAD may be the initial presentation of an underlying vascular disease, vascular specialists should be familiar with the rapidly evolving science of SCAD, including its epidemiology, diagnosis, treatment, and the patient counseling required in this condition.

## Clinical presentation

The clinical presentation of SCAD is similar to MI caused by atherosclerotic coronary artery disease and can encompass all the signs and symptoms of the acute coronary syndrome (13, 14). In one study, 30% of patients presented after physical exertion, and half presented after emotional stress, although it is important to note that no cause of SCAD is found in many patients (14). There is also an association with increased shear stress, such as with exercise, sneezing, or cocaine use (12). Because it may present as unstable angina or even as cardiac arrest, a high index of suspicion is required to make the diagnosis, particularly as patients typically have no or few risk factors for atherosclerotic disease (15).

## Diagnosis

### Coronary angiography

**Figure 1:**
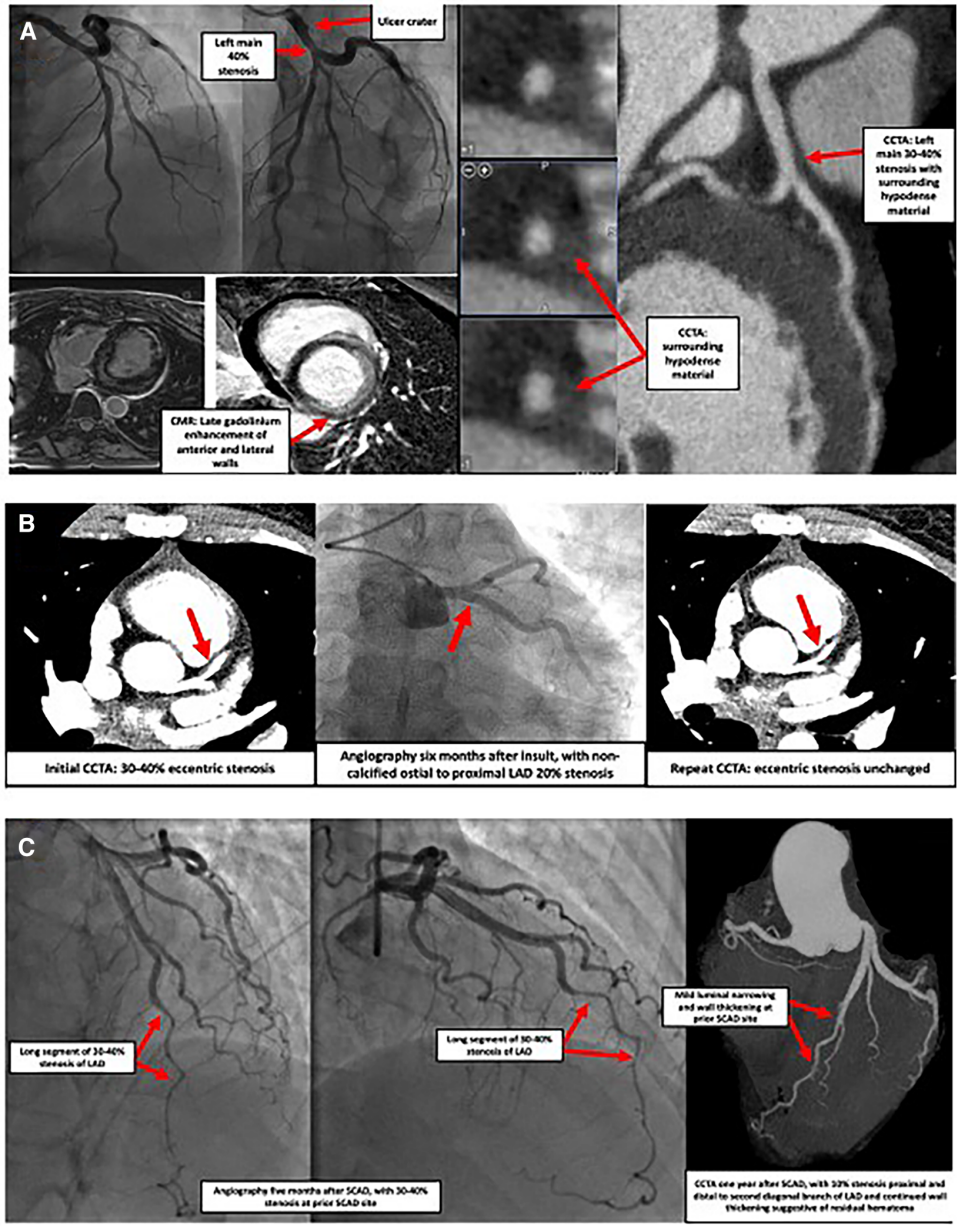
Images of three separate cases showcasing SCAD presentations. (**A**) Case of a 42-year-old female who presented with syncope, ventricular tachycardia, and elevated troponin. Coronary angiography indicated an ulcer crater in the left main coronary artery with 40% stenosis which extended into the origin of the LAD and the LCx (top left panel). Cardiac MRI showed delayed transmural gadolinium enhancement in the anterior and lateral walls as well as the lateral aspect of the inferior wall, suggestive of a vascular insult (bottom left panel). Subsequent coronary CTA found 30%–40% stenosis of the distal left main, with surrounding hypodense material causing vessel enlargement, suggestive of SCAD with intramural hematoma (right panel). Similar findings were seen in the very proximal portions of the LAD and LCx. No atherosclerosis was observed. LAD, left anterior descending coronary artery; LCx, left circumflex coronary artery; MRI, magnetic resonance imaging; CTA, computed tomography angiography; SCAD, spontaneous coronary artery dissection. (**B**) Case of a 30-year-old female with fibromuscular dysplasia who presented with chest pain. CCTA (left panel) showed a 30%–40% stenosis of the ostial to proximal LAD, consistent with a coronary dissection. Coronary angiography (middle panel) and repeat CCTA (right panel) performed six months later showed a focal eccentric 20% ostial LAD stenosis compatible with healed SCAD or intramural hematoma. CCTA, coronary computed tomography angiography; LAD, left anterior descending coronary artery; SCAD, spontaneous coronary artery dissection. (**C**) Case of a 53-year-old female who presented with an acute anterior MI and was found to have SCAD of the mid-to-distal LAD (left panel). Five months later, recurrent chest pain prompted repeat coronary angiography, discovering a focal myocardial bridge and a long region of 30%–40% stenosis at the prior SCAD site (middle panel). No interventions were undertaken. One year after the initial event, another episode of chest pain prompted CCTA, which showed a larger lumen, with only mild 10% stenosis at the prior SCAD site with continued vessel wall thickening suggestive of residual hematoma (right panel). SCAD, spontaneous coronary artery dissection; LAD, left anterior descending coronary artery; CCTA, coronary computed tomography angiography).

While the most commonly affected artery is the left anterior descending artery (LAD) (16, 17), SCAD can affect any coronary artery and is diagnosed via coronary angiography. Four sub-types may be visualized (Table 1) (18). Type 1 is illustrated by multiple lumens and a visible flap. Type 2 is the most common, with abrupt luminal narrowing without flap (Figures 1A,B). Type 3 causes gradual luminal narrowing without a visible flap **(**Figure 1C). Intracoronary nitroglycerin may be helpful to rule out coronary vasospasm as a cause of these various findings, particularly when SCAD types 2 or 3 are suspected (18).

**Table 1:** Classification of SCAD phenotypes by angiography (18).

Classification	Angiographic appearance
Type 1	Multiple lumens visible, with an intervening flap, due to contrast traversing both false and true lumens
Type 2A	Abrupt luminal narrowing without flap, due to intramural hematoma. Stenosis terminates in normal artery.
Type 2B	Abrupt luminal narrowing without flap, due to intramural hematoma. Stenosis continues to end of artery.
Type 3	Gradual luminal narrowing without flap, due to intramural hematoma, often indistinguishable from atherosclerosis. Usually less than 20 mm in length.

**CENTRAL ILLUSTRATION:**
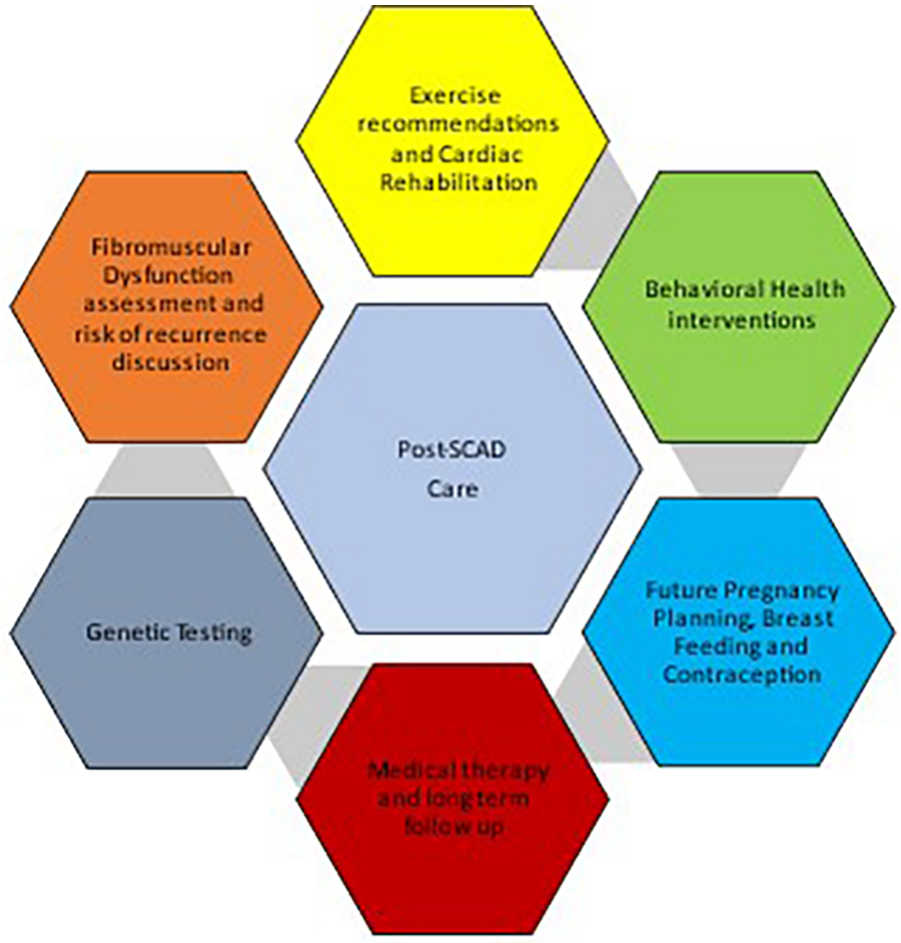
Considerations in post spontaneous coronary artery dissection counseling of patients.

Traditional coronary angiography may not be sufficient to make the diagnosis, particularly when a dissection flap is not visible, such as in type 3 dissection. In these cases, intracoronary imaging with intravascular ultrasound (IVUS) or optical coherence tomography (OCT) is instrumental. These modalities, however, carry a theoretical risk of dissection propagation. However, the benefits of diagnostic precision and precise treatment must be weighed against this theoretical risk. It is important to note that in recent studies on intracoronary imaging in MINOCA, no such complications occurred (19).

### Computed tomography angiography

Coronary computed tomography angiography (CCTA) can reveal intramural hematoma or dissection flap in proximal vessels, as well as perfusion defects, which, if present, can suggest SCAD (20, 21). It is not yet a widely accepted tool, as it cannot rule out SCAD due to resolution limitations, and a case-control study showed that its early use did not reduce death or subsequent MI (22, 23). It may be useful, however, in follow up to clarify if dissected vessels are healing, and in evaluation for anatomic features of the coronary arteries (such as shortened left main and an acute angle between the LAD and the first diagonal branch), which, if present, may cause an increased risk of SCAD due to increased vessel shear stress (24, 25). If CCTA is chosen, protocols with lower doses of radiation should be used as these patients tend to be younger, and more often female (26).

## Epidemiology and pathophysiology

Compared to patients with MI caused by atherosclerosis, patients with SCAD are much more likely to be female, younger, and have fewer risk factors for atherosclerotic disease (14, 17). Almost 90% of patients are female, commonly aged 47–53 years (16, 27, 28). Further, SCAD causes 15%–20% of MI during pregnancy or the peripartum period (3, 29). The reason for this sex predilection is unclear.

SCAD is associated with pregnancy, known as P-SCAD. It affects 1.81 per 100,000 pregnancies, with 70% occurring postpartum (30), usually within the first week (30), and these patients tend to have more severe presentations (30, 31). 1/3 of P-SCAD cases occur in late pregnancy and 2/3 in the early postpartum period (16, 32), and P-SCAD accounts for <5%–17% of SCAD cases overall and 14.5%–43% of pregnancy-associated AMI (2, 14, 18, 33, 34). SCAD has also been associated with multiparity (35–37), clomiphene (35), oral contraceptives (7, 37, 38), and hormone replacement therapy (7, 37, 38). Why it presents often in the peripartum period is unclear, but it could be linked to the hemodynamic changes of pregnancy such as increased cardiac output and blood volume, and reduced systemic vascular resistance (39). It is also possible that perimenopausal/peripartum hormonal fluctuation changes the quantity of elastic fibers and collagen synthesis, as well as the mucopolysaccharide content of arterial walls (3, 18, 31, 35, 39, 40). For example, one study linked estrogen exposure to increased expression of matrix metalloproteinases (MMPs) from vascular smooth muscle cells, leading to necrosis and an unsupported vasa vasorum (41). Additionally, progesterone levels remain high during pregnancy, which can cause loss of normal corrugation of elastic fibers (42). This process could lead to increased susceptibility of rupture with increased hemodynamic stress, as occurs in pregnancy and labor (41).

SCAD is also closely linked with other vasculopathies such as fibromuscular dysplasia (FMD) and has also been described in collagen-elastic fiber disorders such as Ehlers-Danlos and Marfan syndromes (7, 43–46). There is also an inconsistent association with inflammatory disorders which are in general more common in women (12, 30, 47). Regardless of pathophysiology, mortality risk is lower with acute MI from SCAD (in the non-pregnant state) in comparison to MI associated with atherosclerosis (48). This is likely secondary to younger age and lower prevalence of atherosclerotic risk factors (48).

## SCAD and fibromuscular dysplasia

FMD is a noninflammatory, nonatherosclerotic vasculopathy of unclear etiology that is characterized by intima and media hyperplasia, adventitial sclerosis, and destruction of normal elastic tissue. Like SCAD, FMD also primarily affects women. Recent studies have found a significant link between the two conditions (7, 10, 39, 49), especially with concomitant migraines (28, 48). More than 50% of patients with SCAD have FMD, making it the main underlying arterial abnormality observed in SCAD (7, 14, 15, 49). As both conditions are rare, this association suggests causality (39). Because of this strong correlation, all patients with SCAD should undergo screening for FMD with a comprehensive vascular physical examination and with head-to-pelvis CTA (or magnetic resonance angiography, MRA), as finding extra-coronary involvement may affect management and inform prognosis (50). For example, aneurysms or other dissections may require additional treatment to prevent rupture or longitudinal follow up with serial imaging.

Recent advances in genetics have helped to elucidate the link FMD between SCAD. They both appear to be familial in <5% of cases with autosomal dominant inheritance (51). Some studies have revealed common single nucleotide variants, though there is still no explanation as to the sex differences in SCAD (31). The PHACTR1/EDN1 (phosphatase and actin regulator and endothelin 1) locus was the first genetic risk factor to be identified to potentially link SCAD and FMD (52). Further, loss of function of PTGIR (prostaglandin reductase) is enriched in both FMD and SCAD. These mutations seem to lead to non-atherosclerotic arterial stenosis and dissection in patients diagnosed with FMD and SCAD (53).

## Post-dissection care

In this section, we enlist a stepwise format to address and recognize some of the patient facing challenges in the management of post-SCAD care. These concerns focus on rehabilitation of patients and return to functional lives. (Central Illustration, Table 2).

**Table 2:** Post dissection care recommendations.

	Not recommended	Recommended
Exercise	• High-intensity exercise for long periods of time • Competitive and contact sports • Activities performed to exhaustion • Sudden increases in physical activity • Exercise in extreme temperature or terrain • Valsalva maneuver	• Low resistance and high repetition weight training • Conservative approach to exercise mimicking cardiac rehabilitation thresholds
Contraception and preconception counseling	• Hormonal contraception	• Preconception counseling with vascular medicine, cardio-obstetrics, and maternal fetal medicine • Multidisciplinary management throughout pregnancy and postpartum period– maternal-fetal medicine specialists, cardiologists specializing in SCAD, obstetric anesthesiologists
Breastfeeding		• Typically permitted
Genetic testing	• Not typical practice due to low yield	• In cases with high pre-test possibilities via imaging
Cardiac rehabilitation		• Recommended 1–2 weeks post SCAD • Cognitive behavioral therapy should be considered for patients with anxiety or depression • Participation in counseling, support groups, and patient advocacy groups if deemed necessary
Medical therapy	• Dual antiplatelet therapy • Statins • Revascularization, unless the patient presents with high-risk features	• Post event comprehensive vascular imaging • Beta blockers • Antianginal therapy • Conservative management with long term observation

### (a) Exercise

It is generally advised that patients with SCAD should avoid prolonged high-intensity activities, competitive and contact sports, activities performed to exhaustion, sudden increases in physical activity, exercise in extreme temperature or terrain, or performance of the Valsalva maneuver during lifting or exercise (12). Though evidence-based research is lacking, this conservative approach is based on successful clinical practice that mimics the cardiac rehabilitation thresholds previously described (54). Low resistance and high repetition exercises are recommended and deemed safe while weight training (1, 55). The theoretical reason for this advice is that high-intensity activities, sudden increases in exertion, extreme environments, and Valsalva maneuvers may increase arterial shear stress and predispose to recurrence of dissection (36).

### (b) Contraception and preconception counseling

Data are mixed as to whether repeat pregnancy increases the risk of recurrent SCAD (12, 56). However, due to the risk of mortality and significant morbidity associated with a recurrence, these patients should engage in a multidisciplinary decision-making process about the safety of pregnancy. Many clinicians recommend against pregnancy and encourage the use of contraception. If contraception is chosen, hormonal therapy should be avoided due to studies correlating long-term exposure to progesterone and estrogen with an increased risk for SCAD (2, 36). For patients considering pregnancy, vascular medicine, cardio-obstetrics, and maternal-fetal medicine preconception consultation is advised. Not only is there a potential risk of recurrent SCAD with pregnancy, but also there is accumulating evidence that a recurrent episode can have worse outcomes than the first presentation (12). Patients who do become pregnant after SCAD should be followed longitudinally by a multidisciplinary team of maternal-fetal medicine specialists, cardiologists with experience treating SCAD, and obstetric anesthesiologists throughout the prepartum, intrapartum, and postpartum periods. This team should carefully review the patient's prescribed medicines to ensure all medications are safe for pregnancy, conduct baseline cardiac testing, and create a plan for labor, delivery, and postpartum care (12).

### (c) Breastfeeding

Theoretically, hormonal changes during breastfeeding may potentiate the effects of the postpartum period and increase risk for SCAD (57, 58). One study reported that about 3% of women in a cohort were breastfeeding at the time of SCAD (14). However, there is a paucity of data on this specific population, and breastfeeding's potential link to SCAD needs to be investigated further.

### (d) Genetic testing

A thorough family history should be obtained. Genetic testing for connective tissue disorders, however, is typically low yield. In one study, it was diagnostic in only 5% of patients (45). Additionally, familial screening is also relatively low yield, with only 1.2% of individuals being recognized (59). Thus, routine genetic screening is not our current practice, although it can be beneficial and should be performed in some cases with a particularly high pre-test probability (37). For example, if other abnormalities are found on echocardiogram such as aortic root dilatation or aneurysms or other arteriopathy on head-to-pelvis imaging, then genetic testing may be helpful, especially among patients with a strong family or personal history suggestive of vasculopathy.

Recently, there have been more advances that link SCAD patients together with underlying genetic mutations (46, 60). Some of these genetic variants have been linked to other forms of arterial diseases, though SCAD is often the first and only presentation. Variants in genes associated with thoracic aortic aneurysm and dissection (TAAD), Loeys-Dietz syndrome (SMAD2), vascular Ehlers-Danlos syndrome (COL3A1), familial thoracic aortic aneurysm and dissection (LOX), and the cytoskeletal protein talin 1 (TLN) have been reported to have increased prevalence in cohorts of SCAD patients (46, 60).

### (e) Cardiac rehabilitation and psychological care

Patients have reported physical and emotional benefits from participation in cardiac rehabilitation following SCAD, including improvement in chest pain, exercise capacity, psychosocial well-being, and a reduction in repeat cardiovascular events (54). Standard cardiac rehabilitation beginning 1–2 weeks after SCAD has been deemed safe and improves aerobic capacity, body composition, and measures of depression and stress (61). Cognitive behavioral therapy (CBT) is often part of rehabilitation and has been a successful psychosocial intervention for SCAD survivors. It should be considered as part of the treatment plan for patients experiencing anxiety and depression (62). The primary reason for lack of participation in cardiac rehabilitation and structured psychological support is that these programs are not routinely recommended by healthcare providers (63). In addition to cardiac rehabilitation, other avenues of support should also be considered for patients including counseling, support groups, and patient advocacy organizations.

### (f) Medical therapy

#### Antiplatelet agents

One observational study found that at 1-year-follow up, SCAD patients on dual antiplatelet therapy (DAPT) had a higher rate of adverse cardiovascular events, including death, non-fatal MI, unplanned percutaneous coronary intervention (PCI), and bleeding, when compared with single antiplatelet (SAPT) regimens (64). This study was purely observational, but there is still a risk of bleeding with antiplatelet therapies and limited data on long-term benefit. Further, the patients treated with DAPT rather than SAPT may have had more severe presentations of disease. However, despite these limitations, the current evidence does not support dual antiplatelet therapy.

#### Statins

Statins are commonly prescribed after MI (65). However, in patients who are subsequently found to have SCAD rather than atherosclerotic coronary disease, statin therapy is likely not necessary unless the patient meets criteria for a statin for other reasons. Post-event comprehensive vascular imaging (e.g., head-to-pelvis CTA) can be helpful in decision making if atherosclerosis is detected.

#### Beta blockers

Beta blockers are also widely used in the treatment of SCAD, and although routine practice includes the use of beta blockers for SCAD patients, there are no current studies about beta-blocker usage specifically with this population (37). Theoretically, beta blockers may alleviate symptoms, decrease myocardial demand and vessel wall stress, and prevent recurrence (37). These theoretical benefits may indeed be clinically relevant, as a recent study demonstrated markedly reduced SCAD recurrence (hazard ratio 0.36) with beta blockers (66). Beta blockers have the added benefit of reducing the frequency of migraines, which are common among SCAD patients. Counseling patients on possible side effects of beta blockade is important, as fatigue and hypotension are common, which may dramatically affect patients with SCAD, as they are typically young and otherwise healthy without hypertension (12).

#### Antianginal therapy

Patients commonly experience angina after SCAD (30, 54). A careful history and physical examination are essential when a patient presents with chest pain post-SCAD, particularly looking for signs of in-stent restenosis (if stenting was performed) or SCAD recurrence. Physicians should be aware that women are less likely than men to be treated with anti-anginal therapy. Further, in those reporting angina, women were less likely than men to undergo an exercise ECG, to be referred for coronary angiography, to be prescribed antiplatelet and statin therapies, and to be revascularized, even after multivariable adjustment (67). Physicians must be aware of the potential for cognitive bias when treating women and when choosing whether to evaluate angina with advanced imaging studies (68, 69).

##### Role of revascularization

Revascularization with PCI or coronary artery bypass grafting has previously been associated with greater failure rates and major adverse cardiovascular event rates (70, 71). However, a recent meta-analysis concluded that there might not be such a drastic difference in outcomes as previously thought (72). When comparing patients managed with the two approaches, no statistically significant difference in death, MI, revascularization, SCAD recurrence, or heart failure was found. Therefore, conservative management with long-term observation should remain the standard of care unless the patient presents with other high-risk features (71, 73) warranting immediate revascularization such as unremitting chest pain or hemodynamic instability.

## Current clinical trials

Currently, there is one clinical trial specifically looking at the SCAD population. It is a randomized, controlled clinical trial examining the efficacy beta blockers and antiplatelet agents. The primary completion date for the trial is 2024 (74).

## Conclusion

SCAD is a relatively rare form of vascular disease accounting for about 1% of MI and it primarily occurs in women 30–50 years of age. Knowledge of diagnosis and treatment strategies for these patients is crucial for all vascular clinicians. Prompt diagnosis is essential, which should be done with either IVUS or OCT. Non-pregnancy associated SCAD most often occurs in the LAD, while pregnancy-associated SCAD is typically multi-vessel. It is typically treated with antiplatelet therapy and beta blockers, though there is increasing evidence that a more robust combination of post-dissection care is useful. This may include revascularization in rare cases, tailored exercise recommendations, cardiac rehabilitation, and preconception counseling for those considering pregnancy. Clinicians should also be aware of the overlap between FMD and SCAD. SCAD is often misdiagnosed, which can lead to worse outcomes for these patients. Close longitudinal follow-up is essential for patients following SCAD, especially as treatment options continue to evolve. Ongoing clinical trials should shed further light on ideal management strategies in this patient population.
